# Osteoblast role in osteoarthritis pathogenesis

**DOI:** 10.1002/jcp.25969

**Published:** 2017-05-24

**Authors:** Nicola Maruotti, Addolorata Corrado, Francesco P. Cantatore

**Affiliations:** ^1^ Rheumatology Clinic Department of Medical and Surgical Sciences University of Foggia Medical School Foggia Italy

**Keywords:** bone, osteoarthritis, osteoblast

## Abstract

Even if osteoarthritis pathogenesis is still poorly understood, numerous evidences suggest that osteoblasts dysregulation plays a key role in osteoarthritis pathogenesis. An abnormal expression of OPG and RANKL has been described in osteoarthritis osteoblasts, which is responsible for abnormal bone remodeling and decreased mineralization. Alterations in genes expression are involved in dysregulation of osteoblast function, bone remodeling, and mineralization, leading to osteoarthritis development. Moreover, osteoblasts produce numerous transcription factors, growth factors, and other proteic molecules which are involved in osteoarthritis pathogenesis.

## 1 INTRODUCTION

Osteoarthritis is a chronic joint disease characterized by degeneration and loss of cartilage, synovial inflammation, and alteration of peri‐articular bone with osteophytes formation and subchondral bone sclerosis (Davis, Ettinger, Neuhaus, & Hauck, 1988; Valdes & Spector, 2010). These alterations are mediated by cells, such as chondrocytes in the cartilage and osteoclasts, osteoblasts, and osteocytes in the bone. Among these cells, osteoblasts, which are mesenchymal derived cells responsible for bone production and remodeling, regulate skeletal architecture and bone matrix mineralization by producing extracellular matrix proteins, and induce osteoclastogenesis by producing cytokines or by direct cell contact. In osteoarthritis, these cells seem to function differently with a different profile of genes expression. In this review, by considering that osteoblasts dysregulation is involved in numerous bone diseases, we want to focus current knowledge about the role of osteoblasts in osteoarthritis pathogenesis.

### 1.1 Osteoblast differentiation

Osteoblasts are mononuclear specialized cells derived from pluripotent mesenchymal stem cells (Caplan, 1991; Pittenger et al., 1999; Owen, 1988), which can differentiate via activation of different signaling transcription pathways, into different mesenchymal cells lineages, such as osteoblasts, chondrocytes, fibroblasts, myoblasts, and adipocytes (Friedenstein, Chailakhyan, & Gerasimov, 1987; Yamaguchi, Komori, & Suda, 2000).

Among these signaling transcription pathways, a key role in inducing mesenchymal cell differentiation into osteoblast differentiation at an early stage, is played by osterix (Osx) and Runt‐related transcription factor 2 (Runx‐2). Runx‐2 is encoded by Runx‐2 gene, which is also involved in inducing the expression of bone matrix protein genes, such as osteocalcin, osteopontin, type I collagen, and bone sialoprotein (Ducy, Zhang, Geoffroy, Ridall, & Karsenty, 1997; Komori et al., 1997; Miyoshi et al., 1991; Ogawa et al., 1993; Otto et al., 1997). Runx‐2 down‐regulation has been observed in the late stage of osteoblast maturation, when mature osteoblasts form mature bone (Komori, 2010). At a late stage Osx is responsible for inhibiting osteoblast differentiation (Komori, 2003).

### 1.2 The role of osteoblasts in bone metabolism

Osteoblasts are involved in the regulation of bone metabolism by synthesizing bone matrix that becomes progressively mineralized. In fact, osteoblasts are responsible for the deposition of calcium phosphate crystals, such as hydroxyapatite, and produce bone matrix constituents, such as type I collagen. Subsequently, bone matrix progresses into the mineralization phase in which osteoblasts play a role in the production of several proteins, such as sialoprotein, osteopontin, and osteocalcin, that are associated with the mineralized matrix in vivo (Maruotti, Corrado, Neve, & Cantatore, 2012; Neve, Corrado, & Cantatore, 2011). Bone matrix, which is constituted by aligned and ordered collagen fibrils complexed with noncollagenous proteins produced by osteoblasts, is subsequently mineralized via osteoblast regulation of calcium and phosphate local concentrations (Boskey, 1996, 1998).

**Figure 1:**
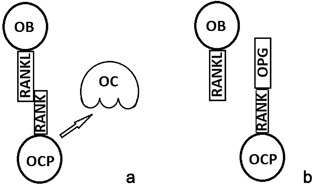
RANKL expressed on osteoblast (OB) mediates a signal for osteoclast (OC) differentiation via binding RANK expressed on osteoclast progenitors (OCP) (a). OPG is a soluble decoy receptor for RANKL, which is involved in the competitive inhibition of RANK/RANKL link, thus avoiding RANK activation and the following osteoclast activation (b)

Moreover, osteoblasts are responsible for osteoclast regulation. Osteoblasts express on their membrane or produce as soluble factor, nuclear factor (NF)‐ĸB ligand (RANKL). The interaction of this ligand, as a consequence of matrix metalloproteinases (MMPs) proteolysis, with RANK, a type I transmembrane receptor expressed on osteoclast precursors, induces osteoclast precursor differentiation into osteoclast (Figure 1a). Subsequently, the RANK‐RANKL complex formation induces the trimerization of RANK and the activation of tumor necrosis factor (TNF) receptor‐associated factor 6 (TRAF6). In turn, TRAF6 is involved in the activation of NF‐ĸB and mitogen‐activated protein kinases (MAPKs), such as p38 and Jun N‐terminal kinase (JNK), which are responsible for the activation of transcription factors such as c‐Src, c‐Fos, and microphtalmia transcription factor (MITF) (Kim et al., 1999; Kobayashi et al., 2001; Matsumoto, Sudo, Saito, Osada, & Tsujimoto, 2000).

Moreover, osteoblasts are involved in the regulation of osteoclastogenesis via modulating RANKL/osteoprotegerin (OPG) ratio (Hofbauer & Schoppet, 2004). In fact, osteoblasts synthesize OPG, a soluble decoy receptor for RANKL, which is involved in the competitive inhibition of RANK/RANKL link, thus avoiding RANK activation and the following osteoclast activation (Figure 1b) (Simonet et al., 1997).

Numerous hormones, including parathormon (PTH), vitamin D, calcitonin, oestrogen, serotonin, and leptin, are involved in the regulation of RANKL and OPG expression in osteoblasts (Neve et al., 2011).

### 1.3 Wnt/β‐catenin signaling pathway

**Figure 2:**
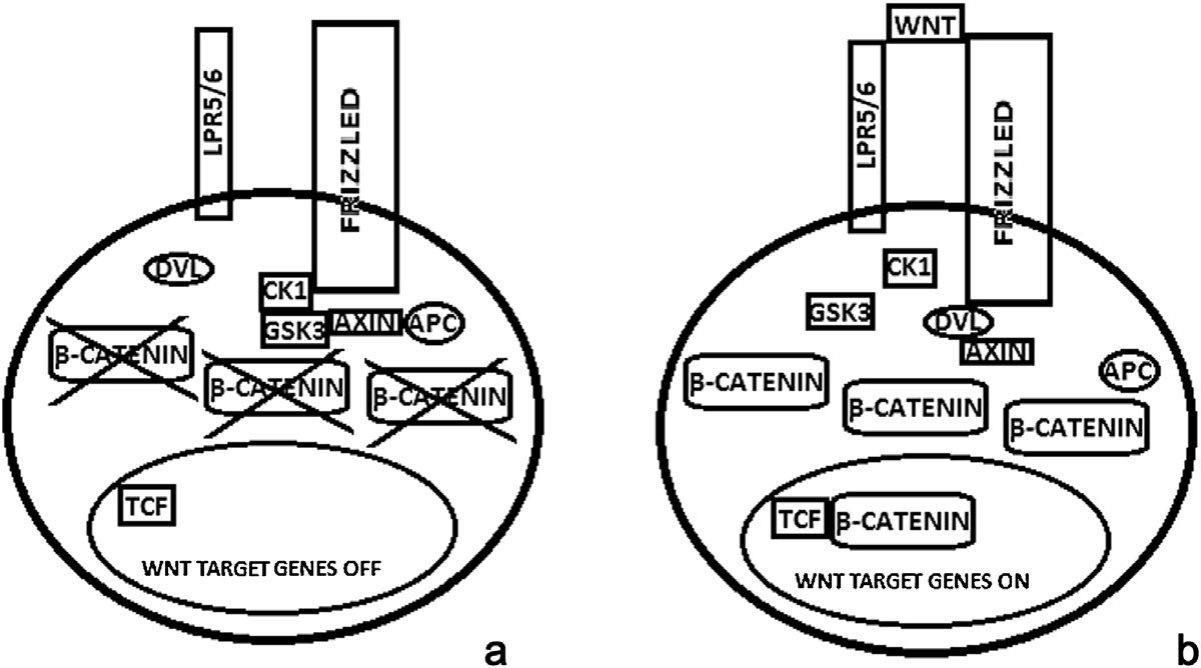
Wnt signaling: at the basal state, GSK3 and CK1 phoshorylate β‐catenin and induce its degradation in the cytosol (a); Wnt binding to LRP‐5/6 and frizzled promotes Dvl‐mediated inactivation of the Axin‐GSK3‐ CK1‐APC complex. Thus, β‐catenin degradation is blocked by avoiding its phosphorylation. Increased β‐catenin levels promote its traslocation into the nucleus, where it forms a complex with T cell factor (TCF) (b). Dvl, dishevelled; GSK3, Glycogen Synthase Kinase 3; CK1, Casein Kinase 1; APC, Adenomatous Polyposis Coli

Several osteoblast biological aspects, such as OPG expression, osteoblast proliferation, and differentiation, are regulated by the Wnt/β‐catenin signaling pathway (Bonewald and Johnson, 2008; Glass et al., 2005; Maruotti, Corrado, Neve, & Cantatore, 2013). The Wnt/β‐catenin pathway is characterized by Wnt binding to its coreceptor complex located at the cell surface, which is composed by the low‐density lipoprotein receptor‐related proteins 5 (LRP‐5) or 6, and a member of the frizzled (Fz) family of proteins (Figure 2a,b) (Tamai et al., 2000; Wehrli et al., 2000). Cytosolic β‐catenin is usually phosphorylated by kinases, such as glycogen synthase kinase 3 (GSK3) and casein kinase 1 (CK1), and constitutes a complex together with Axin and the tumor suppressor adenomatous polyposis coli (APC) (Moon, Bowerman, Boutros, & Perrimon, 2002). Axin plays a role in the phosphorylation of β‐catenin by favoring the union of GSK3 to cytosolic β‐catenin. APC is involved in β‐catenin binding to the ubiquitin‐mediated proteolysis pathway. Wnt binding to the coreceptor induces Frizzled activation and the recruitment of cytosolic Disheveled (Dvl) proteins. Subsequently, Dvl inhibits β‐catenin degradation and induces its accumulation and traslocation, turning it into a nuclear transcriptional regulator responsible for the expression of several target genes (Liu et al., 2002; Maruotti et al., 2013; Willert & Jones, 2006; Yu & Malenka, 2003). Wnt signaling is regulated by secreted frizzled‐related protein family (sFRP) and Wnt inhibitory factor (WIF‐1) (Aberle, Bauer, Stappert, Kispert, & Kemler, 1997), which inhibit the interaction of Wnt with its receptor Fz. Moreover, LRP5/6 activity is antagonized by proteins of the Dickoppf (Dkk) family and by sclerostin, a secreted glycoprotein that is mainly expressed by osteocytes (Westendorf, Kahler, & Schroeder, 2004).

### 1.4 Osteoblasts and osteoarthritis

During last years, an increasing amount of evidences has accumulated in the literature about the role of osteoblasts in osteoarthritis pathogenesis (Corrado, Cantatore, Grano, & Colucci, 2005; Dequeker, Mohan, Finkelman, Aerssens, & Baylink, 1993; El Miedany, Mehanna, & El Baddini, 2000; Hilal, Martel‐Pelletier, Pelletier, Ranger, & Lajeunesse, 1998; Lajeunesse & Reboul, 2003).

There is a close correlation between the OPG/RANK/RANKL system and the subchondral bone alteration observed in osteoarthritis. In fact, an altered expression of OPG and RANKL has been seen in osteoarthritis osteoblasts (Kwan Tat, Pelletier, Amiable, et al., 2008; Kwan Tat, Pelletier, Lajeunesse, et al., 2008)

Two different groups of osteoblasts have been found in osteoarthritis, called low osteoarthritis osteoblasts, which are analogous to normal osteoblasts and are characterized by low levels of prostaglandin E2 (PGE2) and interleukin‐6 (IL‐6), and high osteoarthritis osteoblasts, which are characterized by high levels of PGE2 and IL‐6 (Massicotte et al., 2002). Low osteoarthritis osteoblasts are characterized by decrease in OPG expression and by increased RANKL level, while high osteoarthritis osteoblasts are characterized by increased OPG production and by reduced RANKL expression (Tat et al., 2006). Low osteoarthritis subchondral osteoblasts are characterized by significantly increased membranous RANKL levels compared to normal and high osteoarthritis osteoblasts. Moreover, the treatment with osteotropic factors, such as vitamin D3, IL‐1β, TNF‐α, PGE2, IL‐6, but not PTH, and IL‐17, has been correlated with an increased membranous localization of RANKL on low osteoarthritis osteoblasts compared with high osteoarthritis osteoblasts (Tat et al., 2008). This evidence might explain the different metabolic states of subchondral bone osteoblast subpopulations, with low osteoarthritis osteoblasts probably involved in inducing bone resorption, and high osteoarthritis osteoblasts probably involved in favoring bone formation.

### 1.5 Rate of bone remodeling in osteoarthritis

The rate of bone remodeling is variable across the course of the disease. Early osteoarthritis is characterized by increased remodeling in the subchondral bone, whereas a reduction in bone resorption and an increase in bone formation occur in late osteoarthritis (Findlay & Atkins, 2014). The abnormal bone remodeling and the decreased mineralization are responsible for the altered bone microarchitecture, characterized by increased trabecular number, reduced trabecular spacing, and reduced hardness of the bone (Dall'Ara, Ohman, Baleani, & Viceconti, 2011; Li & Aspden, 1997; Findlay & Atkins, 2014). The extracellular matrix produced by human osteoarthritic subchondral bone osteoblasts is characterized by a reduced amount of minerals and abnormal organization of matrix (Prasadam et al., 2013). The abnormal osteoblast metabolism might also be responsible for the abnormal mineralization of subchondral bone in osteoarthritis. In fact, elevated type I collagen synthesis has been observed in osteoarthritis bone tissue (Mansell & Bailey, 1998). This might be involved in inducing excessive mineralization and subchondral bone sclerosis. In particular, an altered ratio of α1 and α2 chains of type I collagen, with an increased production of the α1 chain, plays a key role in the abnormal mineralization of osteoarthritis bone and may be responsible for the increased levels of transforming growth factor‐β (TGF‐β) in osteoarthritis osteoblasts (Couchourel et al., 2009; Zhen et al., 2013), which is involved in osteophytes production and promotes mineralization via the inhibition of bone morphogenic protein‐2 (BMP‐2) (Neve et al., 2011). Moreover, altered bone remodeling is also involved in osteophytes formation (Findlay & Atkins, 2014). Even if adipokines, including adiponectin, resistin, and visfatin have been associated with the pathogenesis of osteoarthritis, Junker et al. (2016) have recently demonstrated that adipokines do not influence Wnt signaling pathway. Moreover, adiponectin involvement in p38 MAPK signaling activation in osteoblasts has been observed, suggesting that adipokines do not directly influence osteophyte formation but play a proinflammatory role in osteoarthritis (Junker et al., 2016).

### 1.6 Growth factors effects

Osteoarthritis osteoblasts produce the hepatocyte growth factor (HGF) which plays a key role in cartilage loss. HGF is involved in TGF‐β1 expression and inhibits osteoblast response to BMP‐2 (Abed et al., 2015).

As demonstrated in subchondral bone cell culture, high levels of TGF‐β increase levels of DKK‐2, while TGF‐β and DKK‐2 inhibition corrects the abnormal mineralization (Chan et al., 2011).

Moreover, elevated levels of TGF‐β, insulin growth factor‐I (IGF‐I), and IGF‐II have been observed in cultures of human osteoarthritic subchondral bone osteoblasts and in cortical bone explants from the iliac crest of patients affected by osteoarthritis, suggesting an increased biosynthetic activity of osteoblasts (Dequeker et al., 1993; Massicotte et al., 2006).

### 1.7 Ephrins role

Ephrin B4 (EphB4) receptor is highly expressed in low subchondral osteoarthritis osteoblasts, while no differences have been seen between normal and high osteoarthritis osteoblasts in EphB4 receptor expression. Activation of EphB4 receptor by Ephrin B2, inhibits the expression of IL‐1β, IL‐6 and RANKL, but not of OPG (Kwan Tat, Pelletier, Amiable, et al., 2008; Kwan Tat, Pelletier, Lajeunesse, et al., 2008), suggesting that activation of EphB4 by ephrin B2 is involved in the altered subchondral bone metabolism in osteoarthritis by reducing resorption factors levels and function.

### 1.8 Alterations in osteoblast genes expression

The fact that osteoblasts are characterized by an altered function and metabolism in osteoarthritis is also demonstrated by alteration in genes expression which is different from that in either osteoporotic or normal bone. Numerous genes involved in osteoblast function, bone remodeling, and mineralization, such as genes expressing proteins of the Wnt and TGF‐β/BMP signaling pathway, are differently expressed in osteoarthritis (Hopwood, Tsykin, Findlay, & Fazzalari, 2007). Several genes, including soluble Wnts, inhibitors, receptors, co‐receptors, several kinases, and transcription factors, are downregulated in osteoarthritis mesenchimal cells, which include osteoblasts and chondrocytes, during osteogenesis in vitro (Tornero‐Esteban et al., 2015). Several factors involved in Wnt/β‐catenin signaling pathway regulation are dysregulated in osteoarthritis. In fact, reduced levels of Rspo‐2, a Wnt/β‐catenin signaling pathway agonist, and high level of sclerostin, a Wnt/β‐catenin signaling pathway antagonist, have been observed in primary human osteoarthritis osteoblasts cultures (Abed et al., 2011, 2014).

### 1.9 Increased levels of transcriptional regulators controlling osteoblastogenesis

An increased expression of transcriptional factors for osteoblast differentiation, such as RUNX2 and Osx, has been found in bone samples obtained by osteoarthritis patients than compared to osteoporotic patients, suggesting a more intense osteoblastogenesis in osteoarthritis than in osteoporosis (Dragojevič, Logar, Komadina, & Marc, 2011).

Several transcription factors involved in the regulation of the osteogenic lineage have been found dysregulated in osteoarthritis. In fact, by using primary osteoblast cultures from bone samples of osteoarthritis patients, dysregulated expression of TWIST1, TGFβ1, and SMAD3 mRNA has been observed (Kumarasinghe, Sullivan, Kuliwaba, Fazzalari, & Atkins, 2012).

### 1.10 Role of systemic and local factors on osteoblasts

Vitamin D3 treatment has been correlated to a significant increase of osteocalcin in osteoarthritic osteoblasts, proportional to the grade of joint damage (Cantatore et al., 2004; Corrado et al., 2005; Gevers & Dequeker, 1987), suggesting that the altered behavior of osteoarthritic osteoblasts may be related to an abnormal response to systemic or local factors (Cantatore et al., 2004). Moreover, several clinical ex/in vivo and in vitro studies have confirmed the presence of elevated alkaline phosphatase activity and increased osteocalcin levels in primary human osteoarthritis subchondral osteoblasts (Cantatore et al., 2004; Hilal et al., 1998, 2001; Mansell, Tarlton, & Bailey, 1997). On the contrary, no difference in osteopontin levels was found between osteoarthritis and normal osteoblasts (Couchourel et al., 2009). Furthermore, osteoarthritis osteoblasts produce high levels of leptin (Mutabaruka, Aoulad Aissa, Delalandre, Lavigne, & Lajeunesse, 2010). High leptin levels in osteoarthritis might play a role in increased levels of bone markers, such as alkaline phosphatase and osteocalcin observed in osteoblasts (Dumond et al., 2003).

### 1.11 Hypoxia and vascular endothelial growth factor (VEGF) effects on osteoblasts

Alterations of bone vascularization parameters, which may be involved in inducing ischemic episodes associated with hypoxic conditions, have been supposed in osteoarthritis. In fact, hypoxia may stimulate osteoblast production of leptin via hypoxia‐inducible factors (Hif)‐2 regulation, particularly under vitamin D3 stimulation. On the contrary DKK2 is primarily regulated by vitamin D3 rather than hypoxia (Bouvard et al., 2014). Hypoxia also increases the osteoblast production of PGE2, cyclooxygenase 2, angiopoietin‐like 4, type II collagen α1 chain, and insulin‐like growth factor binding protein 1. Moreover, an in vitro study, by using culture of primary osteoblasts isolated from knee bone from patients affected by osteoarthritis, has demonstrated that hypoxia modifies osteoblast phenotype, including the expression of genes that regulate bone matrix, bone remodeling, and bone vasculature (Chang, Jackson, Wardale, & Jones, 2014).

Moreover, osteoblast production of VEGF seems to have a role in osteoarthritis pathogenic mechanisms, as demonstrated by a study on primary human osteoarthritis osteoblast cultures, which showed an increased VEGF expression compared to normal and osteoporotic osteoblasts, both under basal conditions than in the presence of vitamin D3. Moreover, vitamin D3 significantly improved VEGF expression in normal and pathological osteoblasts, suggesting the crucial role of vitamin D3 supplementation in metabolic bone diseases (Corrado, Neve, & Cantatore, 2013).

### 1.12 Endothelin‐1 (ET‐1) effects

Recently, a role for endothelin‐1 (ET‐1) has been proposed in osteoarthritis pathogenesis (Sin, Tang, Wen, Chung, & Chiu, 2015). In fact, ET‐1 is involved in the degradation of articular cartilage in osteoarthritis, and in osteoblast proliferation and bone formation. ET‐1 is responsible for inducing oncostatin M expression in osteoarthritis osteoblasts by trans‐activating the oncostatin M gene promoter via the transcription factor Ets‐1 (Wu et al., 2016).

### 1.13 Osteoblast production of proteases

As suggested by in vitro evidence, altered communications between subchondral bone osteoblasts and articular cartilage chondrocytes may be involved in osteoarthritis pathogenesis, by producing abnormal levels of enzymes, such as A disintegrin and metalloproteinase with thrombospondin motifs (ADAMTS) and MMPs (Prasadam, Crawford, & Xiao, 2012; Sakao et al., 2009; Sanchez et al., 2005).

Moreover, increased levels of MMPs, such as MMP‐13, may also be involved in osteoarthritis cartilage degradation (Sakao et al., 2008). In fact, MMP‐13 is responsible for degrading opticin, a protein typically associated with the extracellular matrix, which plays a role in the structural stability of cartilage. Its cleavage by MMP‐13 may be involved in cartilage degradation (Monfort et al., 2008).

Moreover, lysosomal cathepsins have been reported to play a key role in osteoarthritis pathogenesis and, in particular, cathepsin K has been found highly expressed in osteoarthritis (Logar et al., 2007). Even if cathepsin K is considered as predominantly expressed by osteoclasts, osteoblasts may directly contribute to its production (Mandelin et al., 2006). Nevertheless, the real impact of osteoblast cathepsin K synthesis in osteoarthritis remains to be investigated.

## 2 CONCLUDING REMARKS

Osteoarthritis pathogenesis is still poorly understood and there are not treatment available to repair degraded cartilage and altered bone. Accumulating evidences underline the important role of osteoblasts in osteoarthritis. In fact, osteoblasts are responsible for inducing altered expression of OPG and RANKL, for altering bone microarchitecture by abnormal bone remodeling and decreased mineralization. Moreover, alterations in gene expression are involved in inducing a dysregulation of osteoblast function, bone remodeling, and mineralization, leading to osteoarthritis development. Numerous transcription factors, growth factors, and other proteic molecules involved in osteoarthritis pathogenesis, are produced by osteoblasts. A full understanding of the osteoarthritis pathogenesis could lead to the development of new therapeutic strategies in this disease.

## 3 CONFLICTS OF INTEREST

The authors declare that they have no conflict of interest.
